# Preoperative Aspirin Management in Redo Tetralogy of Fallot Population: Single Centre Experience

**DOI:** 10.3390/healthcare8040455

**Published:** 2020-11-03

**Authors:** Giuseppe Comentale, Gaetano Palma, Valentina Parisi, Silvio Simeone, Gianluca Pucciarelli, Rachele Manzo, Emanuele Pilato, Raffaele Giordano

**Affiliations:** 1 Department of Advanced Biomedical Sciences, Adult and Pediatric Cardiac Surgery-University of Naples Federico II, 80131 Naples, Italy; giuseppe.comentale1990@gmail.com (G.C.); palma.gaetano@libero.it (G.P.); rachele4manzo@gmail.com (R.M.); emapilato@yahoo.it (E.P.); 2 Department of Translational Medical Sciences-University of Naples Federico II, 80131 Naples, Italy; parisi.valentina@tiscali.it; 3 Department of Biomedicine and Prevention, University of Rome Tor Vergata, 00133 Rome, Italy; silviocecilia@libero.it (S.S.); gianluca.pucciarelli@uniroma2.it (G.P.)

**Keywords:** aspirin, redo congenital heart patients, Tetralogy of Fallot, RVOT

## Abstract

*Purpose*: Redo operations and preoperative antiplatelet/anticoagulant therapy can significantly increase surgical risk in congenital heart surgery. This is a retrospective study on the impact of preoperative aspirin therapy on the outcome of Tetralogy of Redo Fallot patients undergoing right ventricle outflow tract (RVOT) conduit implantation. *Methods*: Ten-years retrospective analysis of medical records was carried out. A total of 72 patients were divided into two groups: “Daily-on-ASA” group on daily therapy with aspirin (ASA) until 5 days from surgery and “No-Home-ASA” without it. Propensity match analysis was done in order to standardize the populations. Intraoperative and postoperative lengths were compared as well as the need for inotropic support. In addition, differences in blood transfusions and need for Fresh frozen plasma (FFP)/platelets (PLT) were analysed. *Findings*: Intraoperative lengths were similar between the groups. Not statistically significative differences about postoperative time to extubation (*p* = 0.34), ICU Stay (*p* = 0.31) or in-hospital stay (*p* = 0.36) were found. Drain loss was higher in the “Daily-on-ASA” group (407.9 ± 96.7 mL vs. 349.5 ± 84.3 mL; *p* = 0.03) as well as blood transfusions (372.7 ± 255.1 mL vs. 220.1 ± 130.3 mL, *p* = 0.02) and PLT/FFP need (217.7 ± 132.1 mL vs. 118.7 ± 147.1 mL, *p* = 0.01). No differences were found in postoperative complications or re-explorations for bleeding. *Implications*: We found no advantages in surgical times and hospital stay comparing redo patients who stopped aspirin versus those that didn’t take it in the last 6 months. However, our results suggest that redo patients undergoing RVOT conduit implantation who take daily aspirin are at higher risk of bleeding even if it is stopped 5 days before surgery.

## 1. Introduction

Congenital heart diseases often require a multistage surgical approach but redo operations are burdened by higher risks of morbidity and mortality with an overall standardized mortality ratio of about 8.3% [1,2]. The underlying reasons may lie both in the frequent use of prosthetic materials which, although biocompatible, increase the risk of endocarditis, thrombosis and degenerative phenomena, and the use of antiplatelet/anticoagulant therapies that increase the rate of bleedings [3]. In addition, the patients with complex congenital heart disease during their life often undergo a long series of surgical interventions, many of which need extracorporeal circulation [4]: this technique, although necessary for many of these procedures, is not free from risks since, given the large levels of anticoagulation necessary, it exposes the patient to a high risk of intra- and postoperative bleeding due to the tenacious adhesions that these patients often have [5,6]. These tissues are usually one of the main problems that affect the postoperative outcome of redo patients: as a result of repeated surgical traumas, these structures often modify the mediastinal anatomy and easily expose its organs and vessels to the risk of rupture and to a significant prolongation of the surgery [7]. Despite the frequent use of preoperative imaging to plan the operations, the risk of damaging retrosternal structures such as the right ventricle or the anonymous vein during the median sternotomy remains very high, so much so that it is often preferred to decompress these structures by emptying them through a femoral–femoral cardiopulmonary bypass (CPB) [8].

Due to the above-mentioned issues and the progressive increase in the grow-up congenital heart diseases (GUCH) population, postoperative bleeding in redo congenital heart surgery remains one of the most important problems that the surgeon has to face both during the intraoperative and postoperative period. The lysis of tenacious and vascularized adhesions, the risk of damaging the vascular structures and the sutures on high pressure districts together with the fact that these patients often take antiplatelet and/or anticoagulant therapies create a mix of conditions that dramatically increases the risk of bleeding [9].

It is now universally recognized that non-suspension of aspirin 75 mg (ASA) in adult patients who have to undergo coronary artery bypass surgery seems to not significantly increase bleeding risk or perioperative mortality [10]. However, although referred to adults, the 2017 EACTS (European Association of CardioThoracic Surgery) guidelines on the preoperative management of drugs before cardiac surgery, warn that the withdrawal of ASA in patients at high risk of bleeding such as redo patients should be considered [10].

The lack of evidence on the paediatric population or randomized trials that recommend one rather than another protocol leaves a huge gap about preoperative ASA management in patients undergoing surgical correction of a cardiac malformation. For this reason, each individual centre adopts an its own policy based on their experience.

In order to minimize intra or postoperative bleeding, our center’s protocol usually recommends stopping the ASA 5 days before redo elective procedures that involve right ventricle outflow tract (RVOT). 

In this setting, there are no published data so we retrospectively analysed the outcome of redo Tetralogy of Fallot (TOF) patients on ASA (until 5 days from surgery), and those ones without any antiplatelet/anticoagulant therapy in the last 6 months before RVOT conduit implantation, in order to assess whether preoperative ASA therapy can increase the risk of postoperative bleeding or in-hospital outcomes. 

## 2. Materials and Methods

### 2.1. Design 

Reviewing medical records, we retrospectively analysed preoperative, intraoperative and postoperative data of the 108 redo TOF patients that underwent to a RVOT prosthetic conduit implantations between January 2008 and January 2020 at the Paediatric Cardiac Surgery Unit of the Federico II University of Naples. All patients have previously had at least two cardiac surgeries, including modified Blalock–Taussig palliative shunt (mBT). This was considered among redo operations because it is always performed through a median sternotomy according to the institution protocol.

### 2.2. Participants

**Figure 1:**
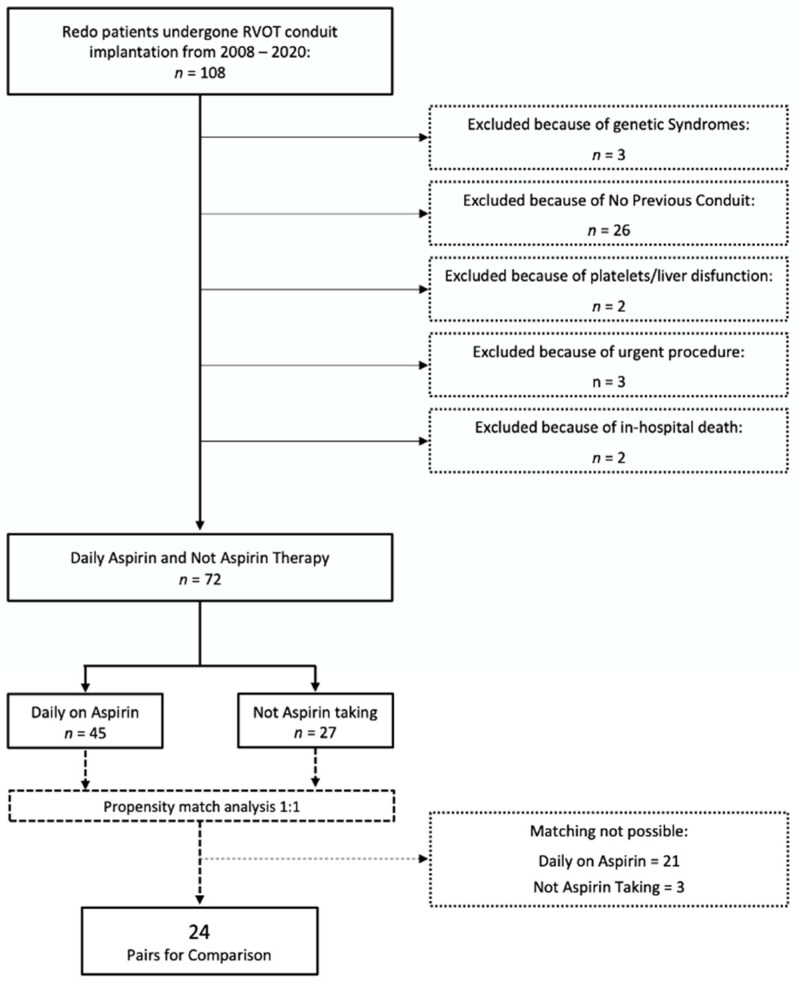
Study Design. RVOT: right ventricle outflow tract

Patients were divided into two groups based on the presence of home therapy with aspirin until 5 days before surgery (Daily-on-ASA). The control group was made up of those patients that were not under aspirin treatment for at least 6 months before the procedure (No-Home-ASA). In accordance to our policy, ASA (dose: 1–5 mg/Kg/day) was prescribed only to patients with prosthetic conduit but the length of this therapy after the surgery was variable and depended of different factors. Many pediatric cardiac surgeons, according to some papers found in the literature, indeed, usually stop aspirin 6 months after conduit implantation. Patients with transannular, infundibular or monocuspid patches were excluded from the study because they do not require postoperative aspirin therapy. Preoperative data (type of heart malformation, age, weight, gender, type of procedure previously done and prosthetic conduit implanted, RACHS-1 Score (Risk Adjustment for Congenital Heart Surgery) [11], comorbidities or urgent procedures), intraoperative data (cardiopulmonary bypass type and time, cross clamping time, type of implanted conduit and topical hemostatic agent used) and postoperative data (hospitalization time in intensive care unit; postoperative drainage; amount of blood products used as concentrated red cells, platelets or fresh frozen plasma; duration of inotropic support; reoperation for bleeding; total time of hospitalization) were recorded. A total of 36 patients were excluded because of the presence of genetic syndromes, congenital platelets deficiency or disfunction, liver failure, right ventricle systolic dysfunction (TAPSE < 12, Tricuspid Annular Plane Excursion), preoperative hemoglobin of less than 12 g/dL or in-hospital death. Thus, the final study population was made up of 72 patients (Figure 1). Of these, the “Daily-on-ASA” group was made up of 45 patients with a prosthetic conduit who received aspirin treatment until 5 days from surgery; on the other hand, the “No-Home-ASA” group was composed of 27 who had a prosthetic conduit on the RVOT and no daily aspirin therapy because it was stopped at least 6 months before surgery. Surgical technique was the same in all 72 patients, with no femoral extracorporeal circulation; in order to prevent postoperative cardiac tamponade, as a centre policy for redo patients, the right pleura was opened, and a drain tube was always put in it. The chosen approach to implant the prosthetic tube on the RVOT, in the absence of residual interventricular or palliative shunts, was always the beating heart technique. The cell-saver blood suction technique was used in all patients. Topical hemostatic agents were the same in the two groups (SURGICEL^®^ Absorbable Hemostat, Johnson & Johnson, New Brunswick, NJ, USA; Tachosil^®^, Baxter, Deerfield, IL, USA). According to the institutional protocol for cyanogen heart diseases, blood transfusion was prescribed in the presence of serum hemoglobin under 10 gr/dL in order to increase it up to 12 gr/dL.

### 2.3. Anesthetic Protocols

Perioperative management was the same for all patients. According to the institutional protocol, surgical anesthesia was obtained through continuous intravenous infusion of propofol/remifentanil for patients older than 7 years and of midazolam/remifentanil for younger children. Before CPB was established, heparin was intravenously administered in a dose of 300 units/Kg in all cases; protamine need, instead, was assessed using the HMS Plus Hemostasis Management system (Medtronic, Minneapolis, MN, USA). 

At the end of surgery, thromboelastography was used for all patients to test the blood coagulation and to verify the need of Fresh Frozen Plasma/Platelets or clotting factors’ concentrates. Postoperative clotting system assessment was performed through ACT (activated clotting time) monitoring during the first 12 h after the operation and through standard clotting analysis (Prothrombin Time, activated Partial Thromboplastin Time, International Normalized Ratio) during the next postoperative days. Protamine was re-administered during the first hours after the surgery if ACT was higher than 140 s or in the presence of active bleeding signs. 

### 2.4. Statistical Analysis

Given the wide variability between the groups and the retrospective nature of the study, in order to minimize statistical errors and to increase power of the study, a propensity match analysis was carried out based on gender, age, weight, RACHS-1 Score, type of the cardiac malformation and surgical technique used (beating heart technique or aortic cross clamping) so 24 matched pairs could be formed (Figure 1). We calculated standardized mean differences for continuous variables and odds ratios (ORs) for categorical variables to check the balance of baseline characteristics. 

Peri- and postoperative characteristics were described by numbers (percentage) or means (±standard deviation, SD). Groups were then compared with unpaired *t*-test or Pearson Linear Correlation for continuous data. We calculated ORs, standardized mean differences and non-parametric difference estimates including 95% confidence intervals as effect measures. A 2-tailed significance level of a = 5% was set for analyses. We performed data preparation and descriptive statistics using IBM SPSS 27 (SPSS Inc., Chicago, IL, USA) and propensity matching using R 3.6.4 (R Foundation for Statistical Computing, Vienna, Austria)

### 2.5. Ethical Consideration

All procedures were in accordance with the ethical standards of the responsible local committee on human experimentation and with the Helsinki Declaration of 1975, as revised in 1983. 

## 3. Results

The global analysis of the 72 consecutive redo TOF patients undergoing RVOT conduit implantation (Table 1) revealed very different, and generally unbalanced preoperative characteristics, in the population that was under daily aspirin treatment until 6 days before surgery (*n* = 45; Mean Age 13.05 ± 4.3; Mean Weight 43.1 ± 14.8; male *n* = 27, 60%) compared to that one who stopped aspirin at least 6 months before the procedure (*n* = 27; mean age 15.07 ± 4.8; mean weight 48.2 ± 16.8; male *n* = 18, 67%). In order to increase statistical power of the analysis and to reduce possible confounding factors, a propensity score matching was carried out obtaining 48 patients for further analysis, divided into 24 pairs (Table 2). 

TOF with pulmonary stenosis was the most frequent heart defect (79.2%) while TOF with Pulmonary Atresia occurred only in the 14% of the cases. Preoperative RACHS-1 score was the same between the two groups. Contegra^®^ conduit (Medtronic, Dublin, Ireland) was the most used RVOT prosthesis both in the Daily-on-ASA Group (*n* = 22, 81.4%) and in the No-Home-ASA patients (*n* = 15, 62.5%). 

Table 3 shows the intraoperative and postoperative results. The mean of CPB time was not statistically different between the two groups (Daily-on-ASA, 97.8 ± 39.6 vs. No-Home-ASA, 103.9 ± 51.2; *p* = 0.64), as well as mean surgical length (453.1 ± 117.9 vs. 455.9 ± 115.1; *p* = 0.93). Hancock^®^ conduit (Medtronic, Dublin, Ireland) was implanted in 31 patients (Daily-on-ASA, *n* = 14 vs. No-Home-ASA, *n* = 17), Contegra^®^ conduit only in 10 patients (6 vs. 4), while fresh homograft was implanted in 7 cases (4 vs. 3).

**Figure 2:**
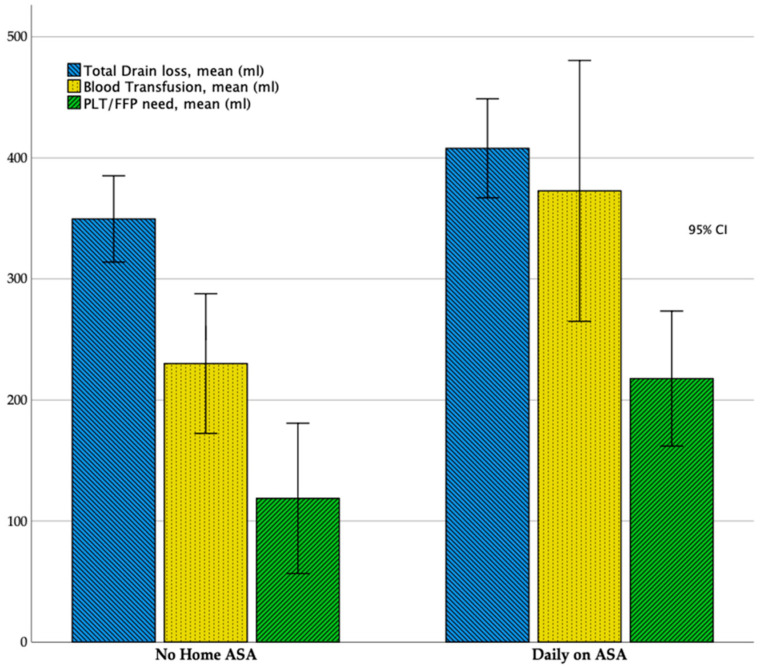
Bar chart showing results of the comparison in Total Drain Loss, Blood and Fresh Frozen Plasma/Platelet (FFP/PLT) transfusions between the two groups. Results are shown as mean value; 95% Confidence Intervals (CI) are reported.

About postoperative data, we observed that, even if much prolonged as absolute value, there were not statistically significative differences among the two groups that may be concerned with postoperative time to extubation (Daily-on-ASA, 28.8 ± 20.9 h vs. No-Home-ASA, 37.1 ± 35.6 h; *p* = 0.34), Intensive Care Unit (ICU) Stay (Daily-on-ASA, 3.8 ± 1.5 days vs. No-Home-ASA, 4.4 ± 2.6 days; *p* = 0.31) and in-hospital stay (Daily-on-ASA, 11.1 ± 4.2 days vs. No-Home-ASA, 12.2 ± 4.2 days; *p* = 0.36). Statistically significant differences were reached comparing the total drain loss and the amount of blood or platelets/fresh frozen plasma (PLT/FFP) transfusions: total drain loss, indeed, was higher in the Daily-on-ASA Group (mean value of 407.9 ± 96.7 mL vs. 349.5 ± 84.3; *p* = 0.03). The mean amount of blood transfusions was higher in patients who were taking daily ASA (372.7 ± 255.1 mL vs. 220.1 ± 130.3 mL, *p* = 0.02) as well as PLT/FFP (217.7 ± 132.1 mL vs. 118.7 ± 147.1 mL, *p* = 0.01) (Figure 2). Inotropic support need was more frequent in the No-Home-ASA group (4 ± 2.8 days vs. 3.1 ± 2.7), although it did not reach statistical significance (*p* = 0.29). There were no differences in terms of postoperative complications (OR = 1.9 (0.51~6.9); *p* = 0.33) and re-exploration for bleeding (OR = 0.95 (0.88~1.04); *p* = 0.31) between the two groups.

## 4. Discussion

Postoperative bleeding or pleural/pericardial effusion in general remains an important cause of morbidity in complex redo paediatric cardiac surgery [12]. These findings are usually attributed to adherences, frequent in redo surgery, and to the length of the surgical procedure and extracorporeal circulation duration. Cardiopulmonary bypass, indeed, has a very high impact on the patient’s body because it causes a severe inflammation response that affects all of the systems, especially the coagulation one [13]. Centrifugal pumping and aspiration forces usually damage the cellular elements of the blood, especially at high rounds per minute. Hemolysis and platelets chelation are usually moderate, and they inevitably have a high impact on intra and postoperative bleeding and clot forming [14]. The hemorrhagic risk can also be intuitively increased by aspirin. Regarding the indication to aspirin therapy in patients with a prosthetic valve on the RVOT, there are conflicting opinions. Tae-Woong Hwang et al. showed that daily aspirin does not give any benefit after 6 months from operation in patients who underwent pulmonary valve replacement with a bioprosthetic valve [15].

In adults, preoperative suspension of aspirin treatment is recommended in those situations in which the risk of bleeding exceeds the benefits related to its continuation (chronic kidney disease, Jehovah’s witness, valvular cardiac surgery, redo operations) [10]. Today, universal consensus maintains preoperative aspirin until the operation only in adult patients with ischemic heart disease where continuation of this drug has been demonstrated to increase short and long patency of the grafts and 30-days mortality compared to suspension. 

In the pediatric population, even if there are no clinical trials or recommendation about perioperative management of aspirin, many centers prefer to stop aspirin 5 days before elective procedures, especially in those patients, such as hypoxemic congenital heart patients, where hypoxia seems to increase the risk of bleeding [16].

Children with chronic cyanosis and congenital heart disease therefore show chronic coagulation system activation and a mild proinflammatory state before surgical procedure but chronic hypoxemia seems to decrease factor VIII:C levels and increase sensitivity to activated C Protein [17,18].

Aspirin treatment, indeed, seems to increase the risk of bleeding because of long-term platelet inhibition [19,20], so a suspension policy before complex and redo congenital heart surgery could be benefitcial in the intra and postoperative management of these patients.

However, there is no evidence regarding a higher bleeding risk even in those patients where aspirin is stopped 5 days before surgery. In the present study, we found that if bleeding seems to be more common in the aspirin group this do not affect the ICU stay or total in-hospital stay but only blood and PLT/FFP transfusions that are more frequent in aspirin group. 

The increased bleeding in the aspirin group did not lead to the requirement of further operations, suggesting that the amount of bleeding was limited and distributed over the ICU stay (407.9 ± 97.6 mL) and not so much in the first postoperative hours, which would have justified surgical re-exploration. No differences between numbers of significant pleural/pericardial effusions 15 days after operation were found, which confirms that the timing in removing the chest drain was accurate.

Preoperative aspirin treatment, in addition, seems to not have any significant impact on surgical or CPB length, which are similar between two groups, indicating that 5-days stopping before surgery is sufficient to guarantee an adequate platelet function and response to protamine during the brief period of the surgery but not during the long period of ICU stay. During the ICU stay, indeed, the difference in bleeding and blood products need among the groups probably reflects a platelet disfunction, which after the first hours after the surgery is masked by the protamine used after CPB. After discontinuation of CPB, indeed, the coagulation system is usually forced to react in order to stop bleeding caused by heparin, and the use of topical hemostatic agents can temporarily reduce surgical bleeding. 

**Figure 3:**
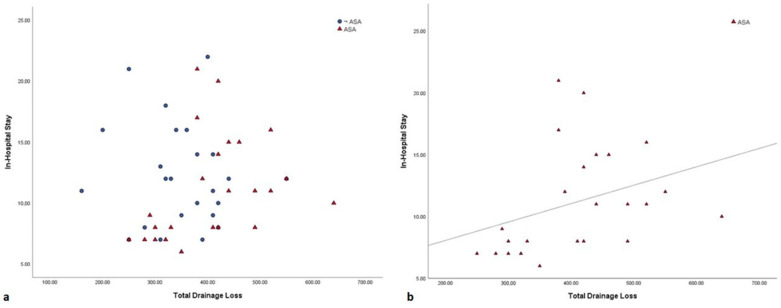
(**a**) Pearson correlation between total drain loss and in-hospital stay in all patients. (**b**) Pearson correlation between total drain loss and in-hospital stay in the Daily-on-ASA group (r^2^ = 0.11).

If we analyse the data about bleeding and hospital stays in the Daily-On-ASA group and then in all patients as one group, we observe that there is a slight positive correlation between postoperative bleeding and hospital stay, indicating that the entity of blood loss drives the hospital stay time (Figure 3). This message should be clearly taken into account because all strategies that can reduce postoperative blood loss have to be adopted, especially in young people where blood transfusion could be strictly avoided [21].

An unexpected result came from the comparison between ICU stay, hospital stay and inotropic support need. Even if differences between the two groups were not significant, the No-Home-ASA group showed a higher hospital stay and a prolonged need for inotropic support (Daily-on-ASA, 3.1 ± 2.7 days vs. 4 ± 2.8 days; *p* = 0.29) compared with the Daily-on-ASA group. In addition, no significant differences among groups about in-hospital infections and non-cardiac complications were found. 

Our study has several clinical and research implications. First of all, it adds to the literature the evidence that a policy of aspirin suspension among elective repeat pediatric patients who had to undergo RVOT conduit implantation is useful and safe because this does not increase the in-hospital stay or surgical length. Further studies are required to clarify whether, among these patients, routinely preoperative platelet function assay could be useful in reducing postoperative bleeding and the need for blood transfusions. However, our study had several limitations, such as it being a retrospective review, and having a small number and heterogeneity of disease entities and bioprosthetic valves. The authors tried to remove any confounding factors in order to improve the statistical significance of the paper but, as is often the case in pediatric cardiac surgery papers, the small number of cases limits the achievement of great statistical power. 

## 5. Conclusions

In conclusion, this study showed that there are no advantages in terms of hospital stay and surgical times when comparing redo patients who stop aspirin versus those that probably did not take it as home therapy: this probably occurs because bleeding is not so high as to impact significantly on hospitalization. However, our results suggest that redo patients undergoing RVOT conduit implantation who take daily aspirin are at risk of bleeding compared to those that are not under aspirin treatment (Figure 3). Aspirin discontinuation 5 days before surgery is probably effective in the reduction in platelets’ anti-aggregation, but it is likely that a quota of dysfunctional platelets remains that is responsible for late bleeding in the postoperative period. Perhaps this kind of patient could be useful for testing routine platelet function before surgery using TEG-PM (TEG Platelet mapping) in order to identify patients at higher risk of bleeding events in the early postoperative period and where to early transfuse a platelet unit after CPB discontinuation [19,22]. We will test this hypothesis in the future. Further research studies are required to guide antiplatelet therapy in this population.

### Take-Home Message

Even if aspirin seems to increase the risk of bleeding, there are no advantages in terms of hospital stay and surgical times when comparing redo TOF patients who stop aspirin versus those that did not take it as home therapy. Preoperative platelet analysis could be a useful tool to identify patients at higher risk of bleeding events in the early postoperative period or that need platelet transfusion after CPB discontinuation.

## Figures and Tables

**Table 1:** Baseline sociodemographic and clinical pre-matching characteristics.

Variables	Daily on ASA (*N* = 45)	No Home ASA (*N* = 27)	*p*-Value
Age, years (mean ± SD)	13.05 ± 4.3	15.07 ± 4.8	0.15
Gender, male (*n*, %)	27 (60)	18 (67)	0.57
Weight, kg (mean ± SD)	43.1 ± 14.8	48.2 ± 16.8	0.18
Heart defect (*n*, %)			
TOF	33 (73.3)	24 (88.9)	0.11
TOF + PAb	1 (2.3)	0	0.43
TOF + PAt	7 (15.6)	3 (11.1)	0.59
Truncus Arteriosus	2 (4.4)	0	-
DORV	2 (4.4)	0	-
RACHS-1 score (*n*, %)			
1 Category	0	0	-
2 Category	34 (82.3)	24 (84.6)	-
3 Category	10 (17.7)	3 (15.4)	-
4 Category	2	0	-
5 Category	0	0	-
6 Category	0	0	-
Aortic Cross Clamp (*n*, %)	6	5	-
Conduit Type (*n*, %)			
Contegra^®^	27 (78.4)	16 (59.2)	-
Hancock^®^	18 (21.6)	11 (40.8)	-
Homograft	0	0	

ASA: Acetylsalicylic Acid; TOF: Tetralogy of Fallot; PAb: Absent Pulmonary Valve; PAt: Atresia; DORV: double outlet right ventricle.

**Table 2:** Baseline sociodemographic and clinical characteristics of 24 propensity-matched patients.

Variables	Daily on ASA (*N* = 24)	No Home ASA (*N* = 24)	*p*-Value
Age, years (mean ± SD)	14.7 ± 3.9	14.4 ± 4.5	0.82
Gender, male (*n*, %)	18 (73.9)	18 (73.9)	1
Weight, kg (mean ± SD)	46.1 ± 11.8	46.3 ± 14.4	0.95
Heart defect (*n*, %)			
TOF	19 (79.2)	19 (79.2)	1
TOF + PAb	0	0	-
TOF + PAt	5 (20.8)	5 (20.8)	1
Truncus Arteriosus	0	0	-
DORV	0	0	-
RACHS-1 score (*n*, %)			
1 Category	0	0	-
2 Category	19 (79.2)	19 (79.2)	1
3 Category	5 (20.8)	5 (20.8)	1
4 Category	0	0	-
5 Category	0	0	-
6 Category	0	0	-
Aortic Cross Clamp (*n*, %)	5 (20.8)	5 (20.8)	1
Conduit Type (*n*, %)			
Contegra^®^	22 (81.4)	15 (62.5)	-
Hancock^®^	2 (8.6)	9 (37.5)	-
Homograft	0	0	-

ASA: Acetylsalicylic Acid; TOF: Tetralogy of Fallot; PAb: Absent Pulmonary Valve; PAt: Atresia; DORV: double outlet right ventricle.

**Table 3:** Intraoperative and Postoperative Results.

Variables	Daily on ASA (*N* = 24)	No Home ASA (*N* = 24)	*p*-Value
Preoperative data			
Surgery length, min (mean ± SD)	453.1 ± 117.9	455.9 ± 115.1	0.93
CPB length, min (mean ± SD)	97.8 ± 39.6	103.9 ± 51.2	0.64
Topical Hemostats, *n* (%)	11 (45.8)	10 (41,6)	0.77
Implanted Conduit (*n*, %)			
Contegra^®^	6 (25)	4 (16.7)	-
Hancock^®^	14 (58.4)	17 (70.8)	-
Homograft	4 (16.6)	3 (12.5)	-
Postoperative data			
Time to Extubation, hours (mean ± SD)	28.8 ± 20.9	37.1 ± 35.6	0.34
ICU stay, days (mean ± SD)	3.8 ± 1.5	4.4 ± 2.6	0.31
Hospital stay, days (mean ± SD)	11.1 ± 4.2	12.2 ± 4.2	0.36
Total Drain Loss, ml (mean ± SD)	407.9 ± 96.7	349.5 ± 84.3	0.03
Blood Transfusion, ml (mean ± SD)	372.7 ± 255.1	220.1 ± 130.3	0.02
PLT or FFP transfusion, ml (mean ± SD)	217.7 ± 132.1	118.7 ± 147.1	0.01
Inotropic Support length, days (mean ± SD)	3.1 ± 2.7	4 ± 2.8	0.29
Complications (*n*, %)	8 (33.3)	5 (20.8)	0.33
UTI	4 (16.6)	2 (8.4)	-
Respiratory	3 (12.5)	1 (4.2)	-
Renal	0	1(4.2)	-
Wound Infection	1 (4.2)	1 (4.2)	-
Re-exploration for bleeding (*n*)	0	1	0.31

CBP = Cardiopulmonary Bypass; PO: Postoperative; ICU: Intensive Care Unit; PLT: Platelet; FFP: Fresh Frozen Plasma; UTI: Urinary Tract Infection; ASA: Acetylsalicylic Acid; **Bold:** statistically significant.
